# Hen-durance training—effects of an exercise regimen on laying hen muscle architecture and fracture prevalence

**DOI:** 10.1098/rsos.241191

**Published:** 2025-02-26

**Authors:** Grace A. T. Hong, Bret W. Tobalske, Nienke van Staaveren, Emily M. Leishman, Tina M. Widowski, Donald R. Powers, Alexandra Harlander

**Affiliations:** ^1^ Department of Animal Biosciences, Campbell Centre for the Study of Animal Welfare, University of Guelph, Guelph, Ontario N1G 2W1, Canada; ^2^ Department of Animal Biosciences, Centre for Nutrition Modelling, University of Guelph, Guelph, Ontario N1G 2W1, Canada; ^3^ Division of Biological Sciences, University of Montana, 32 Campus Drive, Missoula, MT 59812, USA; ^4^ Department Population Health Sciences, Utrecht University, Heidelberglaan 8, Utrecht 3584 CS, The Netherlands; ^5^ Department of Animal Biosciences, Centre for Genetic Improvement of Livestock, University of Guelph, Guelph, Ontario N1G 2W1, Canada; ^6^ Department of Biology, George Fox University, 414N Meridian Street, Newberg, OR 97132, USA

**Keywords:** flapping, physiological cross-sectional area, pectoralis, supracoracoideus, exercise, wing-assisted incline running

## Abstract

Domestic chickens kept for egg laying navigate inclines such as ramps in some commercial housing systems to aid in transitions between housing tiers. Laying hens use their wings and hindlimbs in a locomotion called wing-assisted incline running (WAIR) to ascend steep inclines. There is a potential relationship between the strength of the main flight muscles and the health of the keel bone from which they originate. We sought to test the effects of a controlled, WAIR-based exercise regimen during rearing on keel bone health and muscle properties of white- and brown-feathered laying hens. The WAIR exercise regimen, which consisted of exercise twice weekly for 16 weeks did not promote increases in muscle mass or physiological cross-sectional area at 21 weeks of age (WOA) and did not provide long-term benefits on keel fracture prevalence at 40 WOA. However, the brown-feathered birds were found to have lower amounts of keel fractures at 40 WOA in comparison with the white-feathered birds. Future studies should test for training that begins before chicks become fully feathered, exercises that emphasize full excursion of the wing during downstroke and different levels of intensity, frequency and duration to optimize flight muscle architecture and promote keel bone health.

## 1. Introduction

Incline running, which moves the weight of the body upwards against gravity, is used as a type of strength-training exercise in humans. This type of training challenges not only the cardiovascular system, but also the back and leg muscles [1]. Bipedal ground birds face the challenge of incline running in their natural habitats as they encounter varying terrains [2]. In ground birds, such as guinea fowl and turkeys, the amount of work required by active muscles increases proportionally to the incline [3], where energy expenditure can increase by up to 30% [4]. In both humans and ground birds, the leg muscles produce the majority of the mechanical work during incline running [5]. However, many birds will flap their wings to aid in propelling their bodies upwards and to increase traction on the inclined surface [6]. This is termed wing-assisted incline running (WAIR) in wild birds [6] and in domestic chickens [7]. The addition of wing flapping during WAIR allows ground birds to climb inclines of up to 90° [8].

Domesticated birds kept for egg laying (laying hens) are challenged in aviaries to walk up ramps and master inclines [7,9] as ramps are recommended to aid in transitioning from tier to tier to avoid keel bone fractures [10]. Keel bone fractures are a complex, long-standing concern in the commercial laying hen sector [11]. Mitigating this problem is complicated due to a myriad of contributing factors such as genetics, nutrition and the design of housing systems or equipment [12–15]. The health and physiology of the keel bone and the muscles that attach to it are being explored as key contributors to keel bone fracture incidence [16]. The keel bone of a bird serves as the origin for the main flight muscles; the *pectoralis* (PECT) and *supracoracoideus* (SUPRA). These muscles are the primary ‘motors’, supplying the vast majority of work and power for flight [17]. Although hens may not perform sustained flight like other avian species, these muscles are still used in flapping flight. The effects of controlled exercise on keel bone fractures and the muscles that attach to it have not been extensively studied, but such exercise has been shown to increase take-off velocity [18,19]. As such, the present study begins to address a gap in knowledge relevant to the welfare of layer hens.

Muscles play an important role in protecting against bone injuries and fractures as they absorb shock during impact loading, thereby reducing impact stress on bones [20]. To illustrate, muscle size and strength in humans is inversely related to the susceptibility of bone stress fractures due to overuse [20]. Exercise is demonstrated to positively influence bone health by encouraging higher blood flow and nutrient supply to the bone. Moreover, normal bone loading due to muscle contractions causes bone strains that are sensed by osteocytes which in turn regulate osteoblast and osteocyte activity to maintain healthy bone density for given locomotor tasks [21–24]. Increased bone strength and stiffness, defined by larger bone cross-sectional area, higher bone mineral density and resistance to fractures [25] are positively correlated with long-term, high-impact exercise and physical activity [22].

In laying hens, the PECT and SUPRA drive the wing through downstroke and upstroke, respectively, and are understood to undergo significant stress (force per unit area) and large strain (proportional change in length) to produce work [26]. These main flight muscles are pennated [26], where the muscle fibres insert on an angle to the central tendon [27]. Force produced by a muscle is generally proportional to the physiological cross-sectional area (PCSA) of that muscle [28]. For a given volume of muscle, PCSA is greater in pennated muscles compared with parallel-fibered muscles, therefore pennated muscles generally produce more force than parallel-fibered muscles [27,29]. Thus, increasing the strength or mass of the PECT and SUPRA muscles can significantly improve the quality and health of the keel bone (from which they originate). Enhanced blood flow helping delivers more oxygen and nutrients to muscles and bones, promoting overall health. Additionally, stronger muscles act as better shock absorbers, reducing the stress and risk of fractures on bones.

Herein, we use WAIR as a form of strength training to stimulate the flight muscles in young, growing laying hens to test for a protective effect of this exercise on the keel bone [1,6,30]. As juvenile and adult fowl will typically perform WAIR on steep inclines [7], we exercised birds on a ramp at an incline of 65° over a period of 16 weeks. As strength training may improve bone physiology and function [1], and reduce the risk of fragility fractures in mammals [20], we hypothesized that implementing WAIR during ontogeny will promote muscle hypertrophy and other developmental changes to increase the PCSA of the PECT and SUPRA as an immediate effect. If hypertrophy is observed, we further hypothesized that it would, in turn, reduce the prevalence of keel bone fractures in adult laying hens as a long-term effect. To test these hypotheses, we used a combination of morphometric analysis of the PECT and SUPRA at 21 weeks of age (WOA) and X-ray imaging of the keel bone at 40 WOA.

## 2. Material and methods

### 2.1. Animals and housing

Out of the 129 chicks (*Gallus gallus domesticus*), half were from strain Dekalb White (65 chicks) and half were from strain Hyline Brown (64 chicks). They were randomly assigned to 12 identical floor pens (183 cm L × 244 cm W × 290 cm H) with an equal number of birds from each strain in each pen in a single room from hatch (wing-tagged on hatch day) until they were 40 WOA. An approximately equal number of birds from each strain were present per pen (10–11 female birds per pen; approx. 42.62 cm^2^/bird). Each pen included two platforms (122 cm L × 31 cm W) placed at 92 cm and 74 cm above ground, a high perch (163 cm L × 4 cm W) placed at 181 cm above ground and a low perch (153 cm L × 4 cm W) placed at 15 cm above ground. A feeder, automatic nipple drinkers and three nest boxes were provided per pen. The room was kept at 21°C and a 14:10 h light–dark cycle with a 30 min dawn/dusk period was implemented throughout the experiment.

Each pen was assigned to one of three groups: (i) the WAIR group (exercise training in the form of WAIR), (ii) the HANDLING group (handling only), and (iii) the CONTROL group (no exercise or handling). The groups were distributed throughout the room as evenly as possible. Since the WAIR groups were handled by researchers, the HANDLING group was used to control for any effects that may be due to human handling of birds. Overall, each treatment group consisted of four pens, totalling 43 birds (about an even number of white and brown birds) per WAIR, HANDLING and CONTROL group.

### 2.2. Ramp apparatus

**Figure 1.**
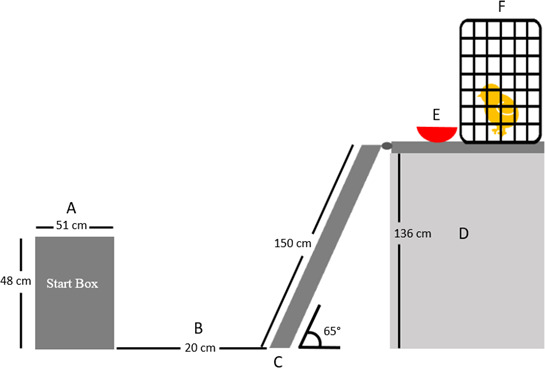
(*a*) Birds were placed in the start box and were allowed 30 s to voluntarily exit. (*b*) Birds walked through a 20 cm walkway to get to the start of the ramp. (*c*) During the exercise training (5–21 WOA, 16 weeks in total), birds ran up the 150 cm L × 10 cm W ramp to get to the top of the ramp apparatus (*d*) which is set at 136 cm for a ramp angle of 65°. (*e*) Birds received corn kernel food reward in a bowl and (*f*) a social reward (conspecifics placed in a crate on the ramp apparatus).

For the WAIR group, a ramp apparatus with adjustable platform heights and ramp inclines was constructed (figure 1). This apparatus included a start box (51 cm L × 31 cm W × 48 cm H) with a sliding door (47 cm H × 15 cm W) which opened to a walkway (width of start box × 20 cm L). A 150 cm length ramp (10 cm width) with an attached platform (70 cm L × 45 cm W) was subsequently inserted onto one of the three shelf heights (115 cm, 130 cm and 136 cm) in the ramp apparatus to create increasing ramp inclines of 50°, 60° and 65°, respectively. These inclines were chosen to ensure use of flight muscles, as chicks and juvenile birds are known to require WAIR to ascend inclines of 50° or steeper [6,7]. The platform that was installed onto one of the shelf heights held a bowl containing a food reward and a crate (45 cm L × 30.5 cm W × 35.5 cm H) containing conspecifics as a social reward. Both the ramp and platform were covered by a 2.5 cm × 1.25 cm wire grid. The ramp was then covered in 60-grit Mastercraft sandpaper and adhesive tape (Duck Tape^®^) in 2.5 cm increments using pieces that measured 4.5 cm L × 10 cm W to provide birds with enough grip to run up the ramp.

### 2.3. Wing-assisted incline running (WAIR) exercise regimen

Prior to chicks becoming fully feathered, they were introduced to the ramp apparatus between 2 and 4 WOA, with the WAIR training administered twice a week for 16 weeks between 5 and 21 WOA. At 1 WOA, chicks were familiarized with a small ramp (81 cm L × 43 cm W × 40.5 cm H) set at a 25° angle in groups of two and rewarded with a social and food reward. At 2 WOA, chicks were introduced to the ramp apparatus described above (figure 1) in pairs at an incline of 50°, which increased to 60° at 3 and 4 WOA, with whole kernel sweetcorn as a special food treat and social rewards to motivate and reinforce birds performing the task. During this introduction period, each chick had two habituation sessions per week where they were required to go up the ramp at least 10 times per session. The WAIR group starting at 5 WOA underwent the exercise regimen twice a week for 16 weeks with at least 1 day of rest in between. At 5 WOA, the young laying hens ran up an incline of 60° until they were 9 WOA. Between 10 and 21 WOA, the incline was increased to 65°. Similarly to the training period between 1 and 4 WOA, the young laying hens were rewarded with whole kernel corn in a bowl and social rewards.

Prior to each exercise event, hens from a single WAIR treatment pen were placed in a crate and individual birds were removed to complete the exercise regimen on the ramp apparatus. Each bird began in the start box (figure 1) and was allowed 30 s to voluntarily exit the start box once the door was opened. If 30 s passed without movement, an observer gently pushed/touched the bird out of the start box. This was required for about 58% of the runs. The bird was allowed 10 s to voluntarily step onto the ramp with both feet. If 10 s passed with no movement, an observer placed the bird onto the ramp. This was required for approximately 70% of the runs. Birds were then required to make their way up the ramp onto the platform where they were rewarded with food treats (sweetcorn) and social rewards (conspecifics). If birds paused on the ramp for more than 5 s, the observer gave them a gentle push to encourage them to make their way to the platform. The amount of time (in seconds) required to ascend the ramp for each run was recorded and used to calculate velocity and track the percentage loss of velocity in real time. After all birds of one pen were tested, the process was repeated with the remaining pens. Pens were paired and tested together on alternating days, alternating morning and afternoon sessions.

Birds were required to individually run up the ramp a minimum of 10 times before the end-of-exercise criteria was considered to be achieved based on Pareja-Blanco *et al*. [31]. Briefly, the WAIR exercise training regimen was considered complete for the day once a 40% decrease in velocity from the fastest run was recorded twice consecutively, two unsuccessful runs were recorded, or a combination of both. Juveniles were not required to continue the WAIR exercise if 5 unsuccessful runs were recorded consecutively, regardless of whether the 10 minimum runs were completed or not. A run was considered unsuccessful if the bird stepped onto the ramp, but voluntarily stepped, jumped or flew off.

### 2.4. HANDLING and CONTROL groups

Like the birds of the WAIR exercise group, entire pens of birds in the HANDLING group were caught and placed in a crate but did not participate in the exercise regime. This procedure was intended to mimic the amount of handling experienced by the WAIR birds. Birds were rewarded with whole kernel corn (food reward used for the WAIR group) once they were returned to their pen.

The birds in the CONTROL group were left undisturbed throughout the duration of the experiment. The CONTROL birds were only handled when taking measurements, such as radiographs. These birds were also given corn at the end of each day to ensure that any increases in mass across the WAIR and HANDLING groups due to the food reward were accounted for in the CONTROL group.

### 2.5. Measurement of the pectoralis and supracoracoideus muscles

At 21 WOA, a subsample of 36 wing-tagged hens (12 from each treatment group with equal number of white- and brown-feathered birds, totalling 3 from each pen) were euthanized by trained staff using CO_2_ and kept in a −26°C freezer for a minimum of 24 h prior to dissection. Dissections took place over 1 week with approximately 4–6 hens dissected per day by one trained researcher who was blinded to the treatments. Whole body carcasses were thawed for an average of 23 h at room temperature. If the carcass was not immediately dissected, it was kept in a fridge at 1°C until time of dissection. Each dissection lasted on average 1.5 h. All carcasses were dissected within 6 h of thawing.

**Figure 2.**
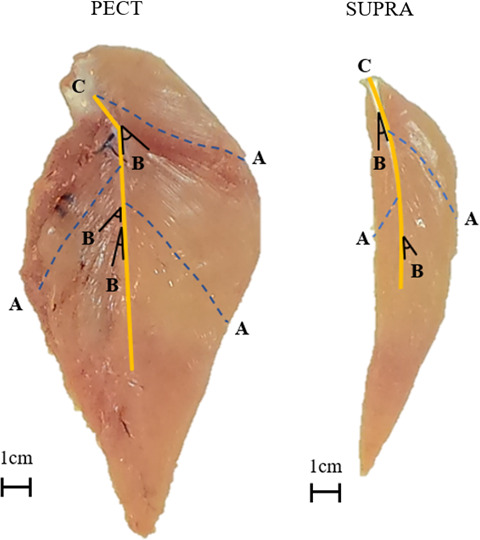
Schematic of muscle measurements shown on the deep side of the pectoralis (PECT) and supracoracoideus (SUPRA) including (*a*) muscle fascicles (dotted blue lines), (*b*) pennation angles (black lines) and (*c*) the central tendon (solid yellow line).

Dissections followed the methods of Casey-Trott *et al*. [32]. Body weight of the whole carcass was taken promptly before dissection using a luggage scale (Maple Leaf Travel Accessories™, ACI Brands, Inc., ON, Canada). The PECT and SUPRA of either the left or right side of the bird was randomly chosen to be removed for analysis. Visible fat and connective tissue on the muscles were removed as much as possible prior to weighing using an analytical balance (Mettler Toledo AE200 Analytical Balance, Mettler Toledo^©^, ON, Canada). The SUPRA and PECT muscles were placed on a piece of cardstock for measuring muscle fibre length (i.e. fascicle length) and pennation angle (figure 2) as described in Hong *et al*. [33]. Ten muscle fibres on either side of each muscle were chosen at random for fascial length (cm) measurement (figure 2*a*). The superficial side was defined as the side of the muscle closest to the surface of the body and the deep side defined as the side furthest from the surface of the body [34]. Ten pennation angles (°, figure 2*b*) were chosen from both sides of the muscle (20 measurements in total per bird) at random and measured using an electronic protractor (Beslands 0−200 mm/8 inches Digital Protractor, Beilong Tool, Zhejiang, China). For the PECT, 1 of the 10 pennation angles measured was assumed to be 0° as the cranial portion of the PECT inserts directly onto the deltopectoral crest of the humerus rather than merging into the central tendon.

Using these measurements and the formula below, the physiological cross-sectional area (PCSA in m^2^) was calculated:


$$PCSA=\frac{M}{p\times L}$$

where *M* represents the mass of the muscle (kg), *p* is muscle density (1060 kg/m^3^ [35]) and *L* is the average muscle fascicle length (m).

### 2.6. Keel fracture measurements

X-ray imaging was used to assess the keel bone fracture status at 21, 30 and 40 WOA. A Poskom VET-20BT portable x-ray unit (Promark Imaging, Toronto, ON, Canada) was used to obtain digital radiographs. Hens were positioned laterally using a custom wooden box with evisceration shackles. A removable wireless DD image receptor panel (29 cm × 24 cm × 3.1 cm, 3.4 kg) was placed approximately 80 cm from the x-ray unit. The height of the panel was adjusted according to the size of each hen so that the panel aligned with the keel bone, allowing the entire keel bone to be radiographed. Hens were gently inverted before their legs were placed into the shackles. Birds remained in this position for approximately 10 s while the radiograph was captured. Image quality and image density parameters of the x-ray unit were 60–80 kVp and 2.5–2.9 mAs.

**Figure 3.**
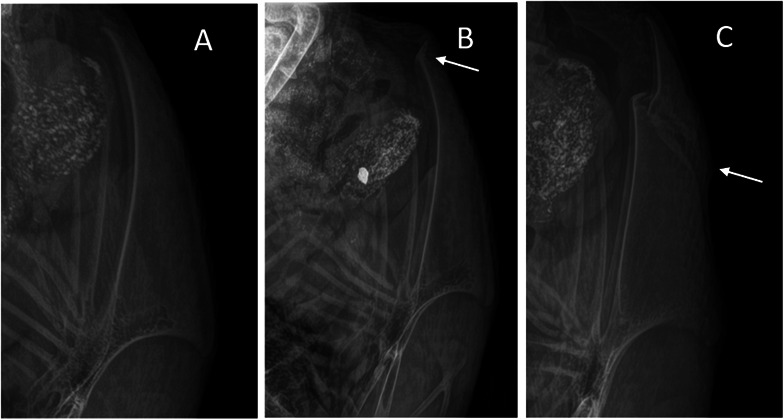
Examples of lateral radiographs taken of a keel bone with (*a*) a score of ‘0’ or ‘no fracture’, (*b*) a fracture score of ‘1.0’ or ‘tip’ fracture where fractures occur close to the caudal end of the keel bone, (*c*) a fracture score of ‘2’ or ‘non-tip’ fracture which includes single or multiple fractures in the non-tip part of the keel and may or may not include tip fractures.

After all radiographs were captured, the presence and severity of keel bone fractures were scored by one trained observer blinded to the training groups. The observer was trained through an online tutorial and used the keel bone fracture severity scale, both of which were developed by Rufener *et al*. [36]. The scoring system was then adapted from the Rufener *et al*. [36] scale to be a better fit for the current data. Radiographs were assigned a score based on a scale of 0–5 with 0.5 increments. A score of 0 indicated no fractures present, while a score of 5 indicated severe fracture(s) affecting the majority of the keel bone (figure 3).

### 2.7. Statistical methods

For ease of interpretation herein, units were converted from kg to g for body mass and from m^2^ to cm^2^ for PCSA. Superficial and deep side data were averaged for muscle mass and PCSA, as no significant differences between the superficial and deep sides were found (*p *> 0.05, electronic supplementary material, figures S1 and S2). Statistical analyses were conducted in SAS On Demand for Academics: SAS Studio v.9.04 (2021, SAS Institute Inc., Cary, NC, USA) using generalized linear mixed model procedures (PROC GLIMMIX) for all dependent variables: muscle mass (g/kg of body weight), PCSA (cm^2^) and keel fractures. Normality and the statistical distributions that best fit the data were determined by assessing studentized residual plots. Results were considered statistically significant if *p*-values were less than 0.05. A Kenward-Roger’s approximation was used to determine degrees of freedom and data presented are least squared (LS) means ± standard error (s.e.).

During dissection and analysis of the PECT and SUPRA muscles, muscle mass (g/kg of body weight) and PCSA (cm^2^) were the two outcome variables obtained. PECT and SUPRA muscle data were analysed separately. To meet normality assumptions, Gaussian distributions were used when modelling the muscle mass of the SUPRA, and the PCSA of both the PECT and SUPRA. A lognormal distribution was used to meet normality assumptions when modelling the muscle mass of the PECT. Fixed effects included training (three levels: WAIR, HANDLING, CONTROL) and strain (two levels: white-feathered, brown-feathered). All models also assessed any interactions that occurred between the fixed effects. Pen was included as a random effect in all models and body weight was included as a covariate in the analyses of PCSA.

As there was insufficient variation in keel fracture scores at 21 and 30 WOA, only keel fracture scores at 40 WOA were analysed statistically. Due to unequal score distributions, keel fracture scores were collapsed into a three-category scoring system (figure 3) with a multinomial distribution. Scores of 0 were considered as no fractures (‘none’). Scores 0.5–2 were classified as ‘tip’ fractures (score 1), as they were found in the caudal tip (cartilaginous portion) of the keel in our study. Higher scores of 2.5–5.0 were classified as ‘non-tip’ fractures (score 2), as they were found cranially in the keel, in the non-tip/non-cartilage part, and may or may not include tip fractures as well. Models were created separately for each strain (white-feathered, brown-feathered) in order to analyse odds of fracture status between treatments, and then, models incorporating both strains were created to analyse odds of fracture status between strains. The odds of fracture status were modelled using training (three levels: WAIR, HANDLING, CONTROL) or strain (two levels: white-feathered, brown-feathered) as a fixed effect and pen as a random effect. Odds ratios are presented with 95% confidence intervals (CI), where an odds ratio > 1 signifies greater risk of a keel bone fracture in comparison with the CONTROL (training) or white-feathered birds (strain).

## 3. Results

### 3.1. Effects of WAIR exercise and bird strain on wing muscle mass and PCSA

Following 16 weeks of WAIR training administered twice a week, there were no significant effects or interactions between the fixed effects (strain: white-feathered, brown-feathered; training: WAIR, HANDLING, CONTROL) in determining the muscle masses or PCSA for either the PECT or SUPRA muscle (*p* > 0.05) (descriptive values in tables 1 and 2). However, there was a tendency for brown-feathered birds (16.1 ± 0.37 g/kg of body weight) to have proportionally larger SUPRA masses compared with their white-feathered (15.1 ± 0.37 g/kg of body weight) counterparts (*F*_1,30_ = 3.31, *p* = 0.0790).

**Table 1.** Descriptive data for body weight (g), muscle fascicle length (cm) and pennation angle (degrees) of the pectoralis (PECT) and supracoracoideus (SUPRA) in white- and brown-feathered laying hens at 21 weeks of age. The averages ± standard deviation (s.d.), maximum (max) and minimum (min) values of the pectoralis (PECT) and supracoracoideus (SUPRA) are presented across the three training groups (WAIR, HANDLING, CONTROL; *n* = 6).

		white-feathered birds			brown-feathered birds		
	muscle	mean ± s.d.	min	max	mean ± s.d.	min	max
body weight (g)							
WAIR		1593.3 ± 95.21	1500.0	1750.0	1655.0 ± 80.93	1560.0	1770.0
HANDLING		1550.0 ± 160.50	1330.0	1680.0	1843.3 ± 171.78	1630.0	2140.0
CONTROL		1571.7 ± 160.80	1310.0	1720.0	1736.7 ± 225.36	1570.0	2170.0
muscle fascicle length (cm)							
WAIR							
	PECT	6.3 ± 0.23	6.1	6.7	6.7 ± 0.30	6.3	7.0
	SUPRA	3.8 ± 0.34	3.4	4.3	4.2 ± 0.43	3.4	4.6
HANDLING							
	PECT	6.4 ± 0.19	6.2	6.6	6.9 ± 0.40	6.4	7.6
	SUPRA	3.9 ± 0.17	3.7	4.2	4.4 ± 0.11	4.4	4.7
CONTROL							
	PECT	6.6 ± 0.46	5.9	7.0	6.7 ± 0.50	6.2	7.4
	SUPRA	4.0 ± 0.31	3.6	4.5	4.0 ± 0.28	3.6	4.3
pennation angle (degrees)							
WAIR							
	PECT	31.3 ± 3.25	27.7	36.4	26.1 ± 4.37	21.0	32.9
	SUPRA	28.0 ± 3.18	21.9	30.5	25.5 ± 3.13	19.4	28.2
HANDLING							
	PECT	26.2 ± 1.07	24.6	27.2	23.7 ± 1.91	21.2	26.0
	SUPRA	26.7 ± 2.71	22.7	29.7	27.6 ± 3.18	24.4	32.7
CONTROL							
	PECT	25.3 ± 3.02	21.6	28.7	24.5 ± 2.21	20.9	26.4
	SUPRA	25.6 ± 2.12	23.0	28.1	27.9 ± 3.69	21.7	31.9

**Table 2.** Descriptive data for the physiological cross-sectional area (PCSA, in cm^2^) and muscle mass (g/kg of body weight) in white- and brown-feathered laying hens at 21 weeks of age. The averages ± standard deviation (s.d.), maximum (max) and minimum (min) values of the pectoralis (PECT) and supracoracoideus (SUPRA) are presented across the three training groups (WAIR, HANDLING, CONTROL).

		white-feathered birds				brown-feathered birds			
	muscle	*N*	mean (s.d.)	min	max	*N*	mean (s.d.)	min	max
muscle mass (g/kg of body weight)									
WAIR									
	PECT	6	46.6 ± 4.10	40.8	50.1	6	48.2 ± 6.41	43.6	59.4
	SUPRA	6	15.1 ± 1.12	13.8	16.8	6	17.2 ± 2.48	14.6	21.2
HANDLING									
	PECT	6	45.7 ± 3.60	41.5	52.0	6	43.0 ± 2.62	38.0	45.1
	SUPRA	6	15.1 ± 1.49	13.7	17.7	6	15.0 ± 1.58	13.26	17.0
CONTROL									
	PECT	6	45.5 ± 2.17	43.3	48.7	6	45.1 ± 3.39	39.2	49.0
	SUPRA	6	15.0 ± 0.78	14.3	16.3	6	15.9 ± 1.57	13.5	18.0
PCSA (cm^2^)									
WAIR									
	PECT	6	11.3 ± 1.40	9.6	13.5	6	11.4 ± 1.28	10.0	13.2
	SUPRA	6	6.0 ± 0.77	5.1	7.17	6	6.5 ± 1.40	5.4	9.1
HANDLING									
	PECT	6	10.5 ± 0.75	9.5	11.6	6	10.9 ± 0.46	10.2	11.4
	SUPRA	6	5.6 ± 0.71	4.9	6.8	6	5.9 ± 0.45	5.4	6.6
CONTROL									
	PECT	5	10.3 ± 0.40	9.7	10.8	6	11.0 ± 0.31	10.6	11.4
	SUPRA	6	5.6 ± 0.48	5.1	6.5	6	6.6 ± 0.79	5.7	7.8

### 3.2. Prevalence of keel fractures

**Figure 4.**
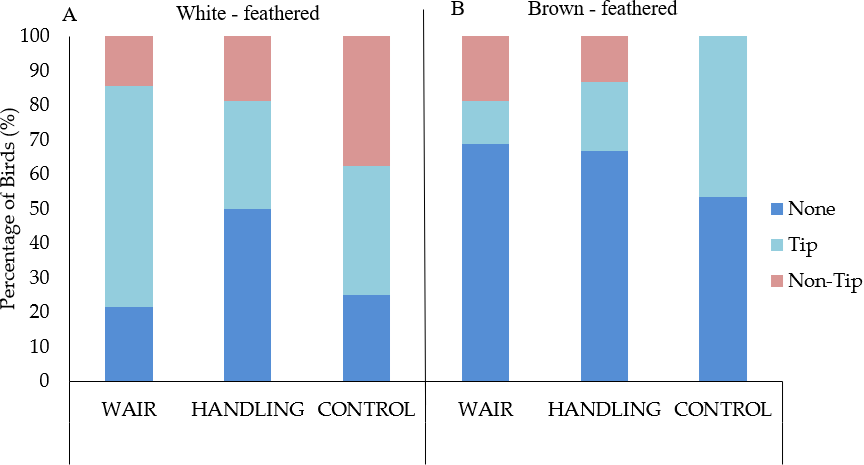
Prevalence (%) of fracture types (none, tip, non-tip) at 40 weeks of age in (*a*) white- and (*b*) brown-feathered laying hens across the three training groups (WAIR, HANDLING, CONTROL). Keel fractures were assessed using the scale of Rufener *et al*. [36] and then categorized into three groups (none, tip and non-tip). Tip fractures include fractures at the caudal end of the keel. Non-tip fractures include fractures on any other part of the keel.

At 40 WOA, 52% of hens showed signs of keel fractures (scores > 0), of which 33% occurred in white-feathered hens and 18% in brown-feathered hens. Tip fractures (35%) were the most common type observed in all groups and in both strains (figure 4). Non-tip fractures (17%) were present in all subgroups, except in CONTROL brown-feathered birds (figure 4).

In white-feathered hens, the WAIR (OR = 0.494, 95% CI 0.038–6.357) and HANDLING (OR = 0.232, 95% CI 0.017–3.133) groups had similar odds of developing keel bone fractures relative to the CONTROL group. Similarly, in brown-feathered hens, the odds of developing keel fracture types did not differ between WAIR (OR = 0.769, 95% CI 0.176–3.354) and HANDLING (OR = 0.769, 95% CI 0.176–3.354) groups when compared with the CONTROL group. However, brown-feathered birds had lower odds of developing fractures compared with white-feathered birds (OR = 0.299, 95% CI 0.130–0.688).

## 4. Discussion

Contrary to our predictions that WAIR training would increase mass and PCSA of the major flight muscles and, in turn, provide protection to the keel, we observed that WAIR exercise, when administered twice weekly, had no significant effects on PECT and SUPRA muscle measurements (mass and PCSA) at 21 WOA or long-term effects on keel fracture prevalence at 40 WOA. It is possible that substantial, immediate effects may be seen if training started earlier prior to chicks becoming fully feathered, or with a more frequently administered/intense exercise. Given the prevalence of keel bone damage in laying hens, the efficacy of alternative exercise regimes merits further study.

### 4.1. Muscle properties of the pectoralis and supracoracoideus

Implementing a 16-week WAIR exercise regimen during the rearing period by requiring the young birds to climb inclines of >50° [7] did not have a significant impact on the muscle mass (g/kg of body weight) or PCSA (cm^2^) of the PECT or SUPRA in either white- or brown-feathered birds at 21 WOA. Previous studies have shown that inclines are more challenging to navigate as it increases the rate of oxygen consumption compared with level-ground walking or running in ground-dwelling birds, such as turkeys and guinea fowl [3,4]. Nevertheless, increases in muscle mass may be more difficult to induce compared with the short-term modulation of metabolic outcomes reported in these studies. Studies by Garant *et al*. [16,37] have shown that decreases in PECT thickness can be induced by wing-feather clipping (mimicking feather loss), as well as wing-immobilization in white-feathered laying hens in a period of 5–6 weeks, whereas birds in this study were exercised twice weekly for 16 weeks without a significant increase in PECT muscle mass. In other studies exploring exercise in broiler chickens, de Almeida Mallmann [38] found that some exercise did not affect the frequency of woody breast (myopathy, characterized by a loss of myofibres and an increase in fibrous tissue, leading to hard tissue see [39]) found in broilers, while Hisasaga and Makagon [40] saw that a more intense regimen where birds exercised daily had positive therapeutic effects. Whether increased frequency or intensity of the WAIR exercise regimen would alter the outcome and whether the current regimen is reflective of daily ramp use basis in a commercial setting needs to be investigated further in future studies. There were also no significant differences between muscle mass of either the PECT or SUPRA between strains, which is surprising as white-feathered birds have been found in previous studies to have larger PECT and SUPRA muscles in comparison with brown-feathered birds when compared on a g/kg of body weight basis [41,42]. However, this could be due to a lower number of birds used in comparison with other studies which used multiple flocks across different farms [41] and housing styles [42].

Strength exercise is defined as any exercise that works the skeletal muscles against an external load (e.g. gravity or lifting weights) with the intent to promote muscle hypertrophy [1]. For this study, WAIR was considered a strength exercise as the young laying hens had to work and pull their own body weight against gravity [1,30]. It is noteworthy that the PECT and SUPRA muscles in birds are theorized to have evolved to output work and power, rather than maximal force [26]. Thus, WAIR may more accurately mimic aerobic or cardio exercise rather than an exercise focused on muscle hypertrophy. Uphill running in ageing humans can help maintain aerobic capacity and cardiorespiratory fitness [43], which can include lowering the maximum heart rate, oxygen consumption and cardiac output [43]. Therefore, the WAIR exercise regimen in this study may have provided alternate benefits in the form of cardiorespiratory fitness and endurance; although this hypothesis requires new tests.

It is likely that hens were not exercising their PECT and SUPRA as intensively during WAIR as they would be during flight. The excursion of the wing at the end of downstroke is less during WAIR in comparison with during free flight [44]. Also during WAIR, the time of wing retraction is slightly prolonged so that it continues into the upstroke [44]. It is possible that the birds are cautious of hitting the ramp with their wings. Direct measures of stress and strain of the PECT of pigeons reveal that motor-unit recruitment, work and power output are less during WAIR compared with free flight [45]. One reason that less power is required from the PECT during WAIR is that the legs are contributing to climbing, integrating the use of legs and wings [6]. Although birds were visibly tired after each exercise bout (e.g. exhibited panting), the WAIR exercise program may have targeted the hindlimb muscles significantly more than the flight muscles. We have a follow-up study presently testing the effects of WAIR on the leg muscles to better elucidate the true impact of WAIR exercise on muscle development.

### 4.2. WAIR and keel fracture prevalence in adult laying hens at 40 weeks of age

WAIR exercise during rearing did not provide protection against keel fracture prevalence in adult life. A study by Casey-Trott *et al*. [32] shows that rearing young laying hens in aviaries, where opportunities for exercise are greater compared with conventional cages, resulted in increased breast muscle mass as well as greater tibia, radius and humerus bone density, mineral content and cross-sectional area. Further results from the study by Casey-Trott *et al*. [32] showed larger keel bones in the birds raised in aviaries and reduced fracture prevalence when subsequently reared in enriched cages in their adult life. Overall, the fracture frequencies in this study are low compared with what has been reported in commercial flocks [11]. In this study, the WAIR group in the present study had a similar chance of developing fractures when compared with the HANDLING and CONTROL groups. It is possible that the floor pen system, which mimics an aviary, used in this study provided easier access to resources compared with commercial multi-tier aviaries. In commercial aviaries, birds would need to navigate vertically higher and potentially steep tiers, especially if there are no navigation aids as platforms, ramps or perches. This could result in more opportunities for injuries to develop, such as fractures. While the results of this study are contrary to our hypothesis, it aligns with the finding of similar muscles masses between exercised and non-exercised groups. Therefore, we cannot rule out that other methods of inducing muscle hypertrophy, such as flapping flight between perches, or a more intensive WAIR training during rearing could reduce the prevalence of keel bone fractures in adult hens.

While the WAIR treatment did not produce significant improvement in overall fracture frequency, significant differences in the type of keel bone fracture were observed between strains. In the white-feathered birds, the CONTROL group had the highest prevalence of non-tip fractures, while the WAIR group had the highest prevalence of tip fractures (figure 4). Since tip-fractures could potentially be associated with the external pressures of perching [14], the WAIR group may have spent more time perching and resting in their pens as birds were visibly tired and exhibiting panting behaviour after their exercises; however, this hypothesis requires further investigation. The pathogenesis of tip and non-tip keel fractures is multifactorial, and the precise mechanisms remain unknown [12]. We can theoretically assume that some non-tip fractures in the CONTROL (non-handled) group may be linked to the birds being more fearful and easily startled during even basic animal care, leading to escape flapping-flight. In wild Galliformes, wing flapping is primarily used to escape threats (46). The increase in volume and/or intensity of escape flapping-flight movements over time could potentially cause overuse stress-fractures, although this remains a theoretical hypothesis. External pressures, such as collisions from uncontrolled landings, are also potential causes of keel fractures [15,47,48]. However, in the current housing system, birds potentially could prevent uncontrolled movements by using their feet as the first and last contact point with the surface, as shown in wild birds to avoid injuries [49]. Furthermore, domesticated birds are able to anticipate locomotion challenges such as inclines/heights [9], and make appropriate choices to avoid such challenges if they have the opportunity [50]. Although not included in this study, future research should include behavioural observations of birds in their home pen to elucidate these potential relationships.

Brown-feathered birds had significantly fewer keel bone fractures at 40 WOA compared with white-feathered birds, consistent with strain differences reported in previous studies [51,52]; however, the results on fracture prevalence between strains can vary across literature [11]. Generally, birds with larger body sizes (i.e. the brown-feathered birds in this study) are less likely to develop fractures compared with lighter birds [52]. In addition, white-feathered pullets and adult laying hens are found to locomote and use elevated resources more often than brown-feathered birds [37,53,54], possibly increasing their chances of collisions that may cause keel fractures. These behavioural differences are attributed to the heavier stature of the brown-feathered birds, which may make them less agile [55]. Taken together, these characteristics could predispose white-feathered birds to developing more fractures due to increased vigorous wing flapping opportunities and/or increased risks of collisions and falls, however this needs to be further explored.

Finally, keel bone fractures can continue to appear and plateau after 45 WOA [15]. As such, differences in keel fracture types (tip versus non-tip) among the treatment groups may only have been detectable if the fracture assessment took place at a later timepoint. Future studies can explore bone breaking strength [56] as a method to evaluate the effects of WAIR training and hen locomotory behaviour on keel bone strength, rather than waiting for fractures to naturally appear with time. This strategy can provide more detail on the mechanics of movement, but requires birds to be terminated, which has ethical implications and was not possible in the current study due to another ongoing project. As an indicator of mineral nutrition and bone mineralization, bone breaking strength has been widely used in studies to look at the strength of long bones in laying hens and broilers [47,57,58]. However, it is not commonly used to assess fracture susceptibility in the keel bone. Bone mineral density may also be an interesting avenue of research in keel fracture studies as human studies demonstrate that bone mineral density can increase with resistance exercises within 16 weeks [59]. In addition, dual-energy X-ray absorptiometry (DEXA) can be used as another method to assess bone mineral content and bone mineral density in laying hens [60].

## 5. Conclusion

The WAIR training applied in the present study from 5 to 21 WOA in fully feathered young hens did not have significant effects on flight muscle mass, physiological cross-sectional area, or confer a protective effect on the keel bone. Brown-feathered hens were found to have a lower prevalence of keel fractures relative to their white-feathered counterparts. These data highlight the importance of incorporating strain differences when designing housing and implementing changes in commercial laying hen farming, as each strain may require personalized accommodations to promote good welfare. Furthermore, other factors such as training that begins earlier in life before chicks become fully feathered, exercise that emphasizes the downstroke, along with variations in intensity, frequency and duration may be crucial for optimizing flight muscle architecture and promoting keel bone health.

## Data Availability

Data and code have been uploaded as electronic supplementary materials [61].
